# Publisher Correction: Cortical dendritic activity correlates with spindle-rich oscillations during sleep in rodents

**DOI:** 10.1038/s41467-017-01652-8

**Published:** 2017-11-23

**Authors:** Julie Seibt, Clément J. Richard, Johanna Sigl-Glöckner, Naoya Takahashi, David I. Kaplan, Guy Doron, Denis de Limoges, Christina Bocklisch, Matthew E. Larkum

**Affiliations:** 1 NeuroCure Cluster of Excellence, Charité-Universitätsmedizin, D-10117 Berlin, Germany; 2 Surrey Sleep Research Centre, University of Surrey, GU2 7XP Guildford, UK; 3 Bernstein Center for Computational Neuroscience Berlin, Humboldt-Universität zu Berlin, D-10115 Berlin, Germany; 4 Institute for Biology, Humboldt-Universität zu Berlin, D-10117 Berlin, Germany; 5 Department of Physiology, Universität Bern, 3012 Bern, Switzerland

*Nature Communications* **8**:684 doi:10.1038/s41467-017-00735-w; Article published online 25 September 2017

In the originally published version of this Article, incorrect references were cited on two occasions in the Results section. Under the subheading ‘Ca^2+^ activity in single dendrites and somata of L5 neurons’, the final sentence of the second paragraph incorrectly cited reference 29 instead of reference 31. Under the subheading ‘Spiking of L5 cell bodies is not influenced by spindles’, the first sentence cited reference 30 instead of reference 29. These errors have now been corrected in both the PDF and HTML versions of the Article.

