# Supplementary material for: Cortical dendritic activity correlates with spindle-rich oscillations during sleep in rodents
Source: Nat Commun. 2017 Sep 25;8:684. doi: 10.1038/s41467-017-00735-w (PMC5612962; doi:10.1038/s41467-017-00735-w)
Supplement: Supplementary file 1 — Supplementary InformationSupplementary Figures and Supplementary Table [file 41467_2017_735_MOESM1_ESM.pdf]

File name: Supplementary Information
Description: Supplementary Figures and Supplementary Table

File name: Peer Review File

SUPPLEMENTARY FIGURES

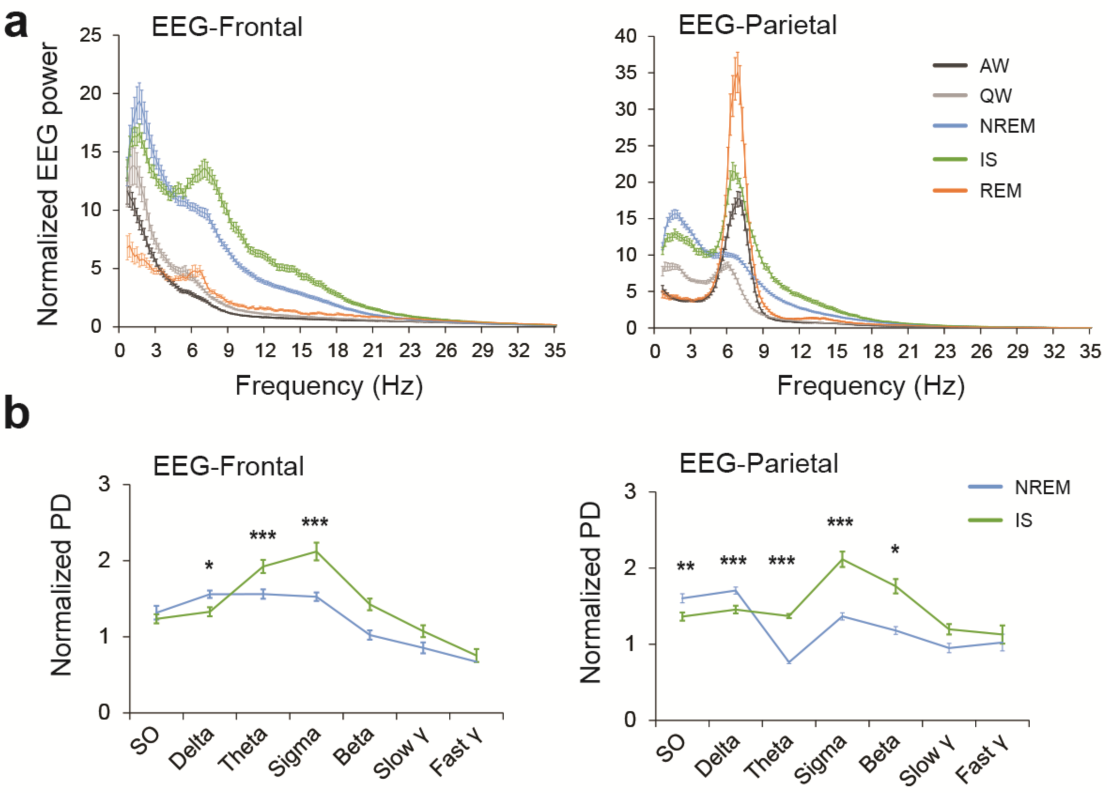

Supplementary Fig. 1 EEG changes across behavioural states (a) Average EEG power spectra for frontal and parietal EEGs for all five behavioural states. **(b)** Comparison of PD for several frequency bands between NREM and IS sleep (****P* < 0.001, ***P* <0.01, **P* <0.05, t-test). Data from all groups were pooled due to similar trend (two-way ANOVA; factor "*group*": *P* > 0.8 for all frequency bands in frontal and parietal EEGs across behavioural states, *n* = 28). All values represent means ± s.e.m.

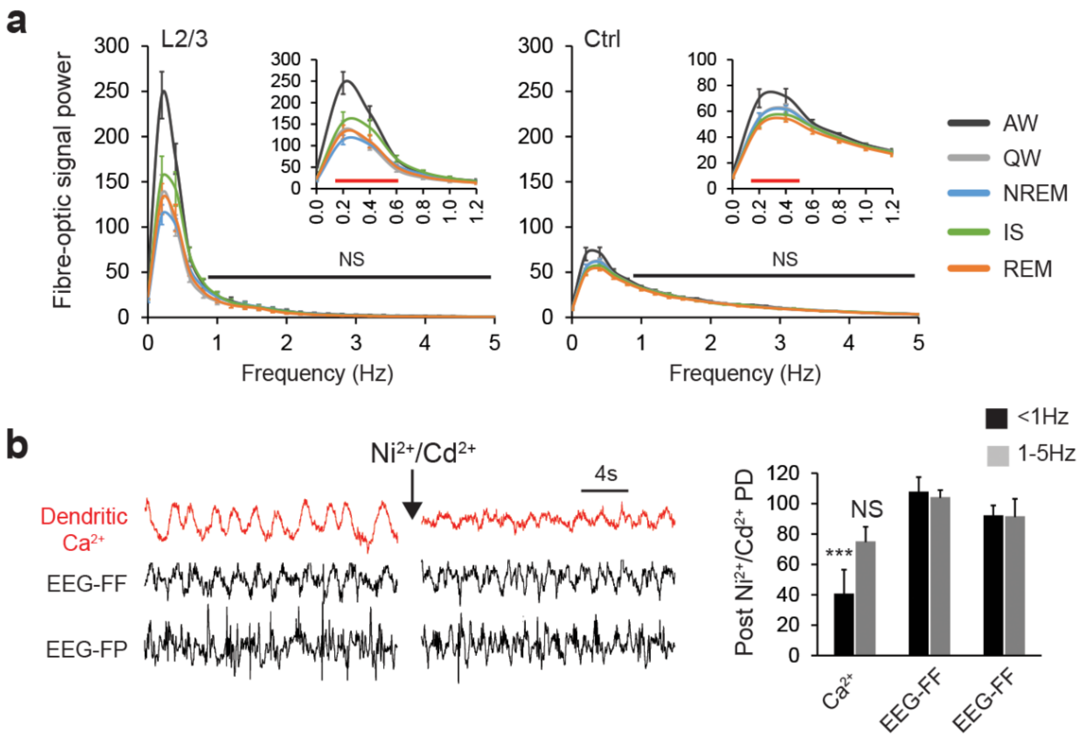

Supplementary Fig. 2 Validation of the 0.1-1Hz PD frequency band for fibre-optic Ca^{2+} activity measurements (a) Average power spectra of the fibre-optic signal (normalized to the mean across behavioural states) recorded in L2/3 (n = 11) and Ctrl (n = 6) groups. Significant differences between states were only found for frequencies < 1Hz (two-way ANOVA; factor "*state*"; L2/3: $F_{4,1248}$ = 16.85, Ctrl: $F_{4,650}$ = 11.85; P < 0.001 for both groups; red line (*inset graph*): P < 0.05, black line (*main graph*): P > 0.05, Holm-Sidak test). **(b)** Blocking Ca^{2+} currents in dendrites with Ni^{2+}(2µM)/Cd^{2+} (1µM) in anaesthetized animals (n = 8) significantly decreased (~60%) the Ca^{2+} signal recorded for oscillations < 1Hz but not > 1Hz (baseline vs. post-drug application: <1Hz, t = 7.29, P < 0.001; 1-5Hz, t = 1.24, P = 0.24, t-test). Drug application did not affect EEGs in either frequency band (baseline vs. post-drug application: FF (<1Hz), t = 0.48, P = 0.88; FF (1-5Hz), t = 0.09, P = 0.93; FP (<1Hz), t = 0.75, P = 0.47; FP (1-5Hz), t = 0.72, P = 0.48, t-test). All values represent means ± s.e.m.

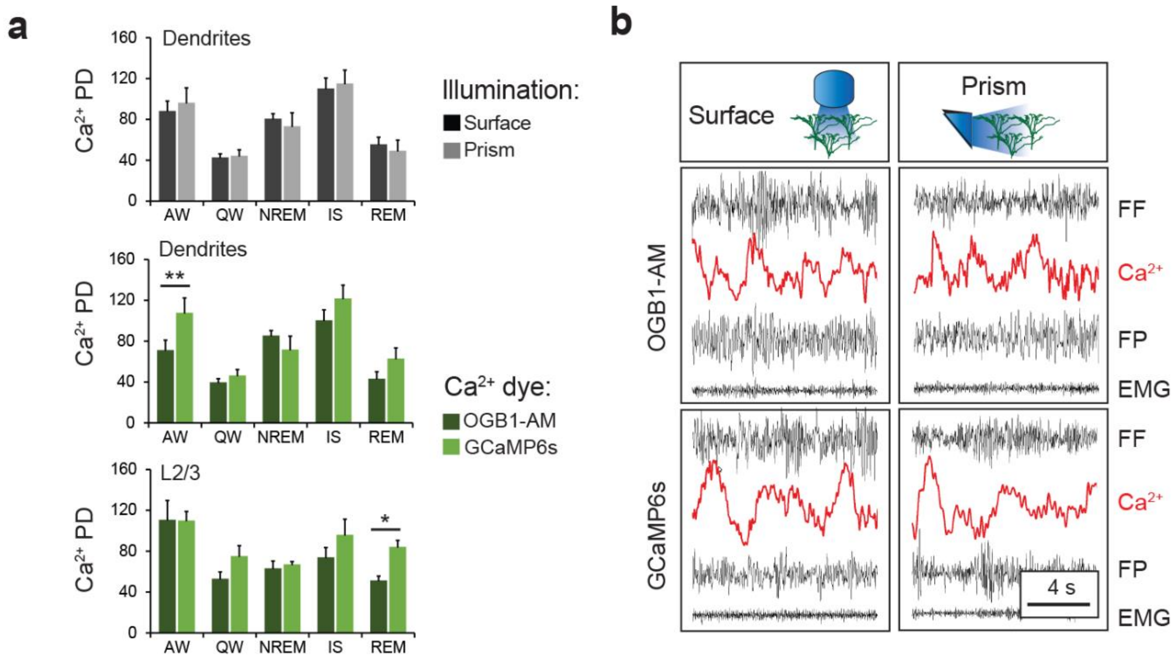

Supplementary Fig. 3 Ca^{2+} changes across brain states are independent of Ca^{2+} dye or illumination angle (a) Changes in the dendritic Ca^{2+} PD do not depend on the illumination angle (three-way ANOVA; factor "*illumination*": $F_{4,44} = 0.001$, $P = 0.98$; *upper graph*). There was, however, a significant trend towards higher values for recordings with GCaMP6s compared to OGB1-AM in both groups (factor "*dye*": L2/3, $F_{4,44} = 6.12$, $P = 0.02$ two-way ANOVA; Dendrites, $F_{4,44} = 8.55$, $P = 0.005$ three-way ANOVA; $**P < 0.001$, $*P < 0.05$, Holm-Sidak test, *lower two graphs*). These differences were significant during AW in dendrites and REM sleep in L2/3. The main results reported in Figure 2c are therefore not affected by this effect. Values represent means ± s.e.m. Number of animals for each group are reported in Supplementary Table 1. **(b)** Examples of dendritic Ca^{2+} signals recorded during IS with different combinations of dye and illumination angle. EEGs (FF & FP) and EMG signals are represented.

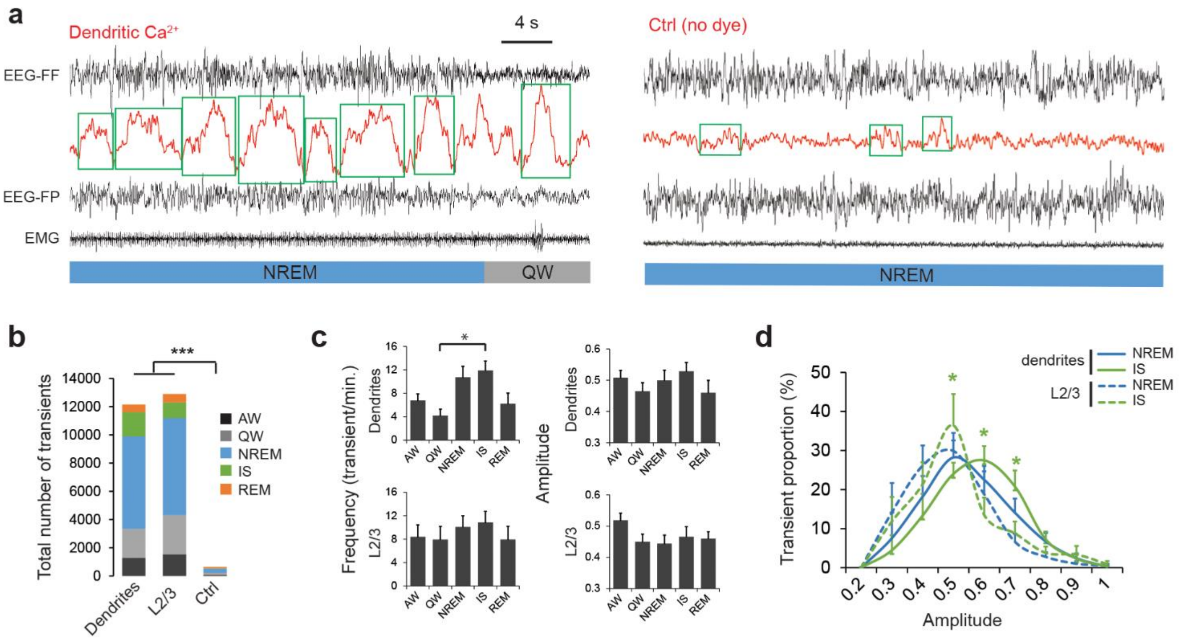

Supplementary Fig. 4 Ca^{2+} transient analysis (a) Segments of combined Ca^{2+}/EEG recordings showing examples of detected transients from population of dendrites (*left*) and from a rat that did not express any Ca^{2+} indicator (Ctrl, *right*). EEG/EMG are in black, optical recordings are in red and behavioral states are represent at the bottom of each recording segment. **(b)** Cumulative number of transients across behavioral states in each group (two-way ANOVA; factor "*group*": $F_{2,125} = 10.27$, $P < 0.001$; vs. Ctrl, *** $P < 0.001$, Dendrites vs. L2/3, $P = 0.75$, Holm-Sidak test). The number of transients detected in each behavioural states were comparable between dendritic and L2/3 recordings ($P > 0.5$ for all states, Holm-Sidak test). **(c)** Ca^{2+} transient frequency (*left graphs*) and amplitude (*right graphs*) for dendritic and L2/3 recordings (*Frequency* (dendrites): $H = 13.64$, $P = 0.009$ Kruskal-Wallis one-way ANOVA on Ranks; *$P < 0.05$, Dunn's test). **(d)** Mean percentage of transients of a given amplitude (represented in 0.1 bins) in NREM and IS (expressed as percentage of all transients/animal) for dendritic and L2/3 recordings (*group* X *interval* during IS: $F_{8,180} = 2.68$, $P = 0.008$ one-way ANOVA; *$P < 0.05$, Dunn's test). For (c) and (d) values represent means ± s.e.m. $N = 11$ for dendrites and L2/3 groups and $n = 6$ for the Ctrl group.

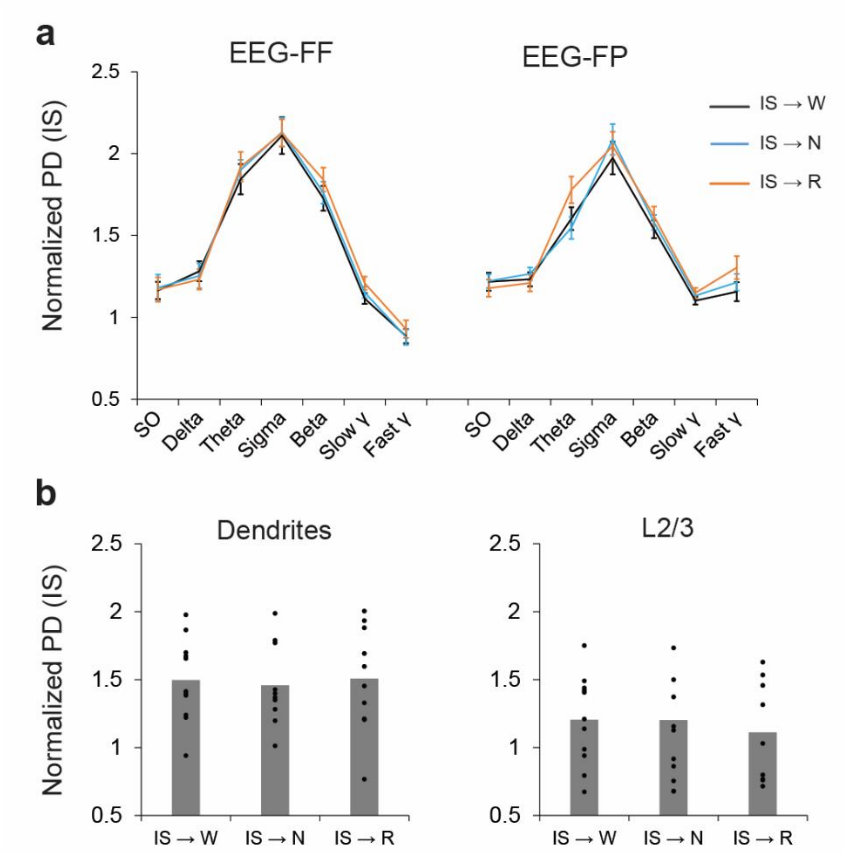

Supplementary Fig. 5 EEG and Ca^{2+} activity at IS transitions (a) IS oscillatory signature (i.e. PD frequency distribution in frontal and parietal EEGs) were similar at IS-WAKE (W: AW/QW), IS-NREM (N), and IS-REM (R) transitions (two-way ANOVA; factor "*transition*"; EGG-FF: $F_{2,406}$ = 1.04, *P* = 0.35; EEG-FP: $F_{2,406}$ = 2.07, *P* = 0.13). Values for both groups (dendrites & L2/3) were pooled (*n* = 22) and represent means ± s.e.m. **(b)** Mean (grey bars) Ca^{2+} activity in dendrites (*n* = 11) and L2/3 neurons (*n* = 11) did not vary depending on IS transitions (Dendrites: $F_{2,28}$ = 0.06, *P* = 0.94 one-way ANOVA; L2/3: H = 0.63, *P* = 0.73 Kruskal-Wallis one-way ANOVA on Ranks). Black dots = individual animals. For (a) and (b), a single value (average EEG PD or Ca^{2+} PD) for each type of transition was calculated for each animal.

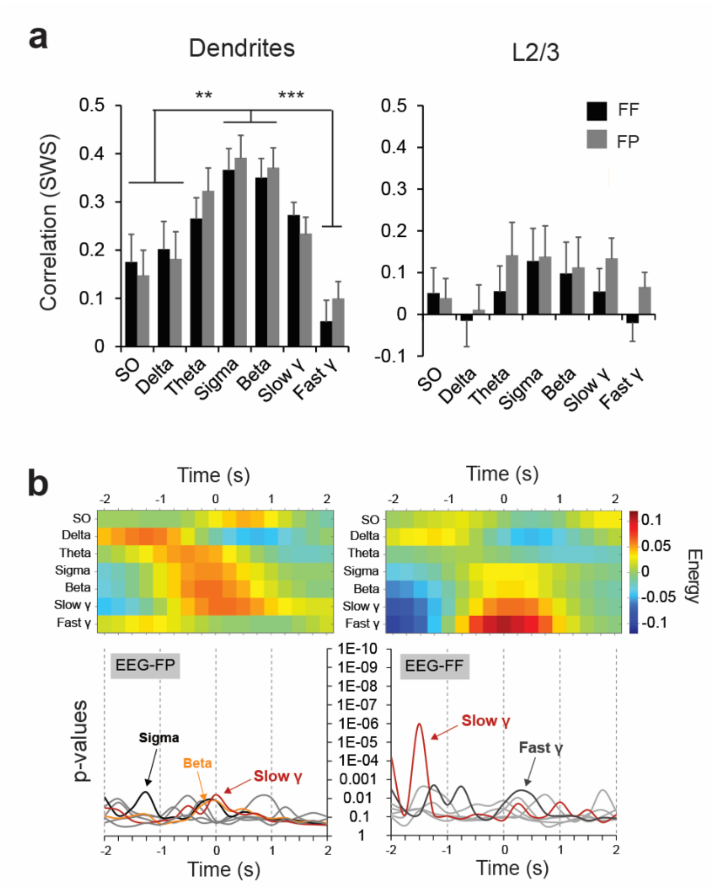

Supplementary Fig. 6 Relationship between Ca^{2+} activity in population of dendrites and L2/3 neurons and EEG during SWS (a) Mean (± s.e.m.) correlation (Pearson) coefficient across animals between Ca^{2+} and EEG PDs for all frequency bands for dendrites (*left graph*) and L2/3 (*right graph*). Values from individual 4-s epochs were used for correlations (mean number of SWS epochs: dendrites = 991.18 ± 83.16, L2/3 = 1029.1 ± 94.15, *n* = 11/group). Two-way ANOVAs showed no significant effect of EEG (Dendrites: $F_{1,140} = 1.15$, $P = 0.7$; L2/3: $F_{1,140} = 1.57$, $P = 0.21$) but a significant effect of frequency bands only for dendritic recordings (Dendrites: $F_{6,140} = 11.69$, $P < 0.001$, L2/3: $F_{6,140} = 1.27$, $P = 0.27$; *** $P < 0.01$, ** $P < 0.01$, Holm-Sidak test). **(b)** Time-frequency analysis for L2/3 recordings. Results for EEG-FP (left) and EEG-FF (right) are represented. Energy heat maps (*upper graphs*) with corresponding *p*-values (*bottom graphs*) for a ± 2-s cross-correlation time window (see Methods).

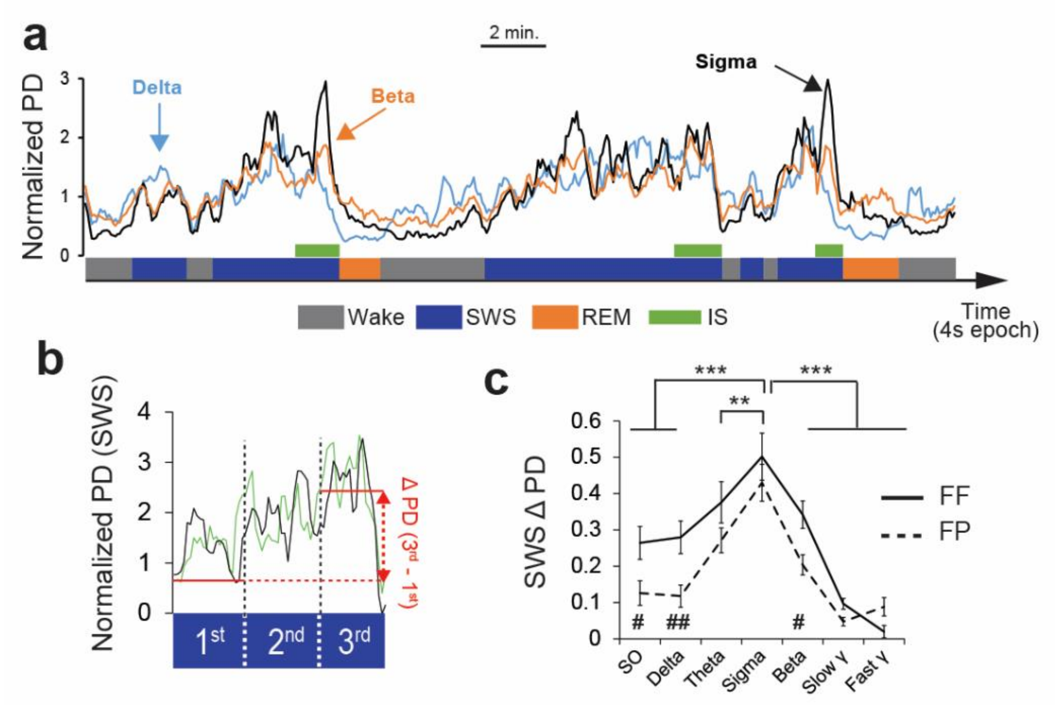

Supplementary Fig. 7 Network oscillatory dynamics during SWS (a) Example of EEG power (PD) fluctuations for delta, sigma and beta bands across behavioural states. Data are represented as trendlines (Methods). **(b)** Illustration of the ΔPD (PD 3rd – PD 1st) calculation used for (c). **(c)** A three-way ANOVA showed no significant effect of group (Dendrites vs. L2/3: $F_{1,280} = 1.51$, $P = 0.22$) but a significant effect of both EEG ($F_{1,280} = 16.41$, $P < 0.001$) and frequency bands ($F_{1,280} = 26.83$, $P < 0.001$). Mean (± s.e.m.) ΔPD during individual SWS episodes is largest in frontal areas and for sigma power compared to other frequency bands (FF vs. FP: ## $P < 0.01$, # $P < 0.05$; vs. sigma: *** $P < 0.001$, ** $P < 0.01$; Holm-Sidak test, $n = 22$ [dendrites & L2/3]).

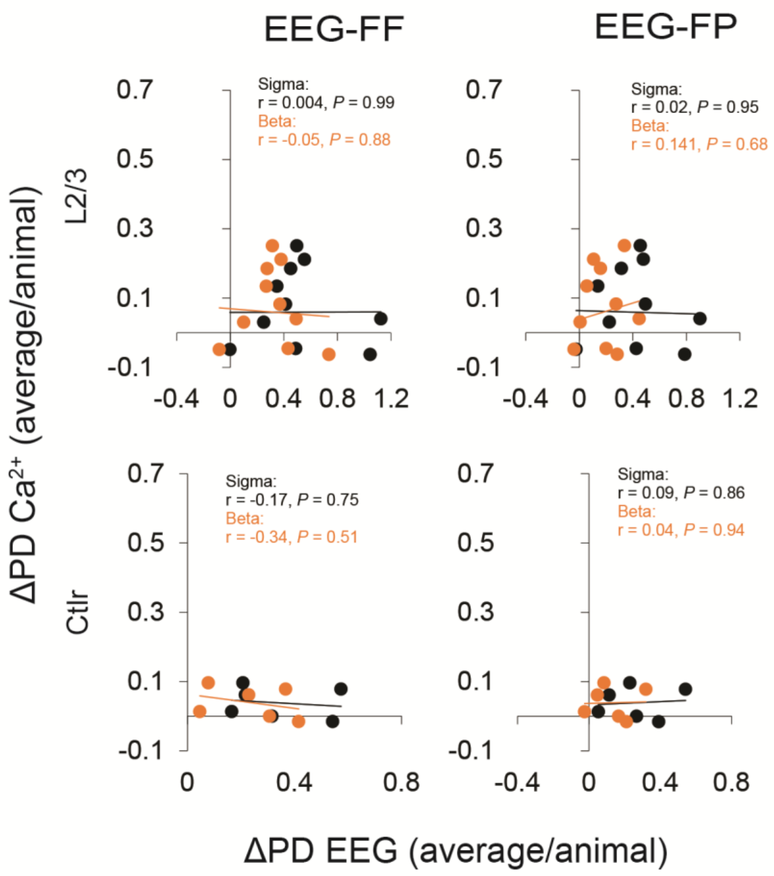

Supplementary Fig. 8 Correlation between the magnitude (ΔPD) of Ca^{2+} and sigma-beta PDs changes during SWS episodes for L2/3 and Ctrl recordings Scatter plots showing the correlation (Pearson) coefficient (and associated *P* values) between the average (across SWS episodes/animal) Ca^{2+} and sigma and beta PDs for frontal (FF) and parietal (FP) EEGs (values represent individual animals, *n* = 11 for L2/3 and *n* = 6 for Ctlr).

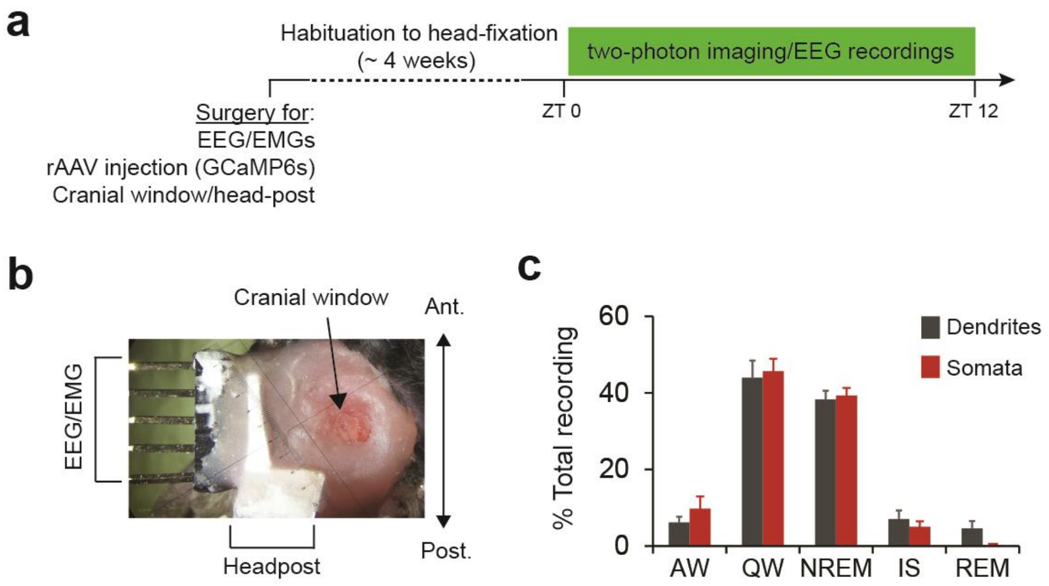

Supplementary Fig. 9 Experimental design and behavioural states for two-photon experiments **(a)** Experimental design. **(b)** Head implant for combined two-photon Ca^{2+} imaging and EEG/EMG recordings. **(c)** Average (mean ± s.e.m.) time spent in each behavioral state did not differ between dendritic and somatic recordings (two-way ANOVA; factor "*group*": $F_{1,20}$ = 2E-05, P = 0.99; n = 3/group).

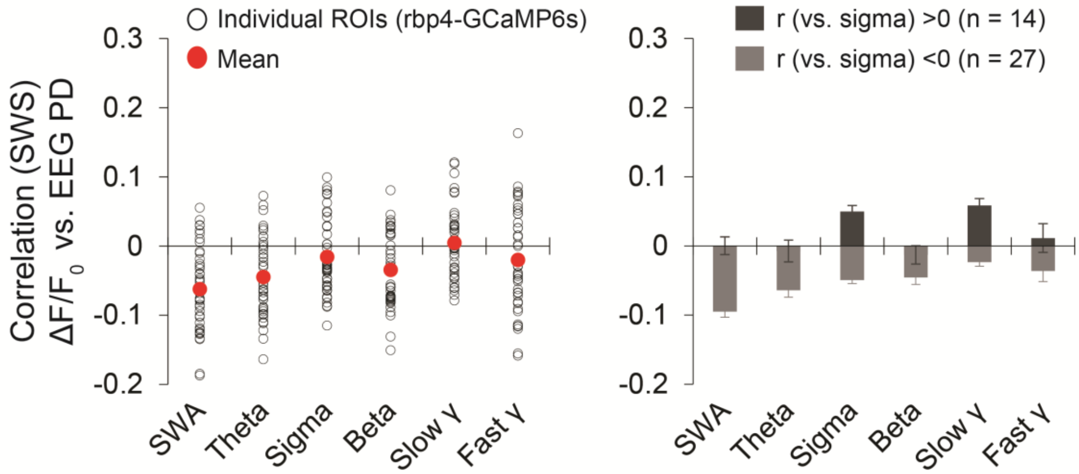

Supplementary Fig. 10 Correlation between Ca^{2+} activity in individual soma of L5 pyramidal cell and EEG during SWS The Rbp4-cre mouse line was used to express GCaMP6s (floxed-AAV) specifically in L5 pyramidal neurons. (*Left panel*) Correlation between ΔF/F0 for all single somata (*n* = 41 cells in 1 mouse) and EEG-FP PDs for different frequency bands during SWS (SO and delta bands were combined into slow wave activity [SWA: 0.5-4Hz] due to similar trends). Values from individual 4-s SWS epochs were used for correlation. (*Right panel*) Mean (± s.e.m.) correlation across somata selected to have a positive (r>0) or negative (r<0) correlation with sigma PD.

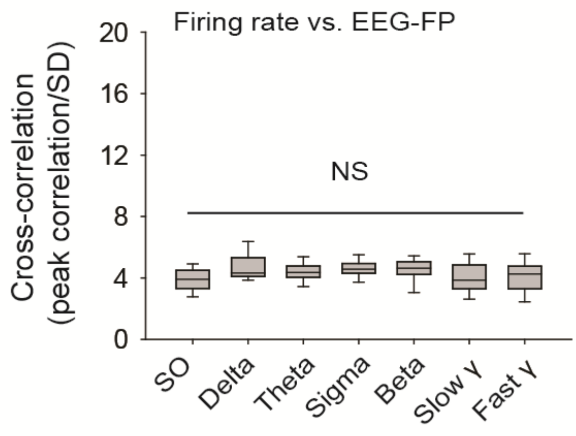

Supplementary Fig. 11 Cross-correlation between L5 cell bodies firing rate and parietal EEG frequency bands Similar cross-correlation analysis as for the frontal EEG (Fig. 7e). Post-hoc comparisons following the two-way ANOVA revealed no significant difference between frequency bands for parietal EEG but a significant lower correlation of firing rate with delta oscillations compared to frontal EEG (Delta: EEG-FF vs. EEG-FP, $P < 0.05$, Holm-Sidak test). Values represents means ± s.e.m.

SUPPLEMENTARY TABLE

**Injected layer**	**Illumination**	**Dye**	**N**	**Age**	**Analyzed time (min.)**
**L5**	Surface	OGB	4	38.5 ± 0.98	127.3 ± 30.43
	Surface	GC	3	46.3 ± 3.48	144.38 ± 21.9
	Prism	OGB	1	32	162.33
	Prism	GC	3	43 ± 2.52	156.07 ± 21.78
**L2/3**	Surface	OGB	5	37 ± 0.71	142.99 ± 13.38
	Surface	GC	6	42.67 ± 1.31	130.39 ± 14.42
**Ctrl**	Surface	none	3	35.67 ± 0.67	184.29 ± 12.94
	Surface	Flx.GC	3	47 ± 1.53	158.6 ± 23.64
Combined					
**L5**			**11**	**41.27 ± 1.72**	**142.99 ± 13.04**
**L23**			**11**	**39.67 ± 1.26**	**131.15 ± 10.06**
**Ctrl**			**6**	**41.33 ± 2.64**	**171.44 ± 13.35**

Supplementary Table 1 Groups for freely behaving experiments The table presents number (N) and mean age of rats in each group and the duration of the recorded session used for analysis (*Analyzed time*). We also represent the distribution in each group of animals that were recorded using similar imaging conditions (illumination [Surface vs. 90° angled "Prism"] and Ca^{2+} dye [OGB1-AM (OGB), GCaMP6s (GC) and Flex-GCaMP6s [Flx.GC]). There was no difference in age ($F_{2,25} = 0.18$, $P = 0.84$ one-way ANOVA) or analyzed time ($F_{2,25} = 1.84$, $P = 0.18$ one-way ANOVA) between groups. All values represents means ± s.e.m.
